# Item Analysis of the Behavior Rating Inventory of Executive Function‐Adults Version Using the Rasch Model in Two Forms for People With Schizophrenia in Taiwan

**DOI:** 10.1002/mpr.70034

**Published:** 2025-08-11

**Authors:** En‐Chi Chiu, Ya‐Chen Lee, Yi‐Ching Wu, Shu‐Chun Lee

**Affiliations:** ^1^ Department of Long‐Term Care National Taipei University of Nursing and Health Sciences Taipei Taiwan; ^2^ Department of Occupational Therapy College of Medical and Health Science Asia University Taichung Taiwan; ^3^ Department of Occupational Therapy Taipei City Psychiatric Center Taipei City Hospital Taipei Taiwan; ^4^ Department of Recreation and Sports Management University of Taipei Taipei Taiwan

**Keywords:** caregiver, executive functions, Rasch analysis, schizophrenia

## Abstract

**Background:**

The Behavior Rating Inventory of Executive Function‐Adults version (BRIEF‐A) consists of two forms, self‐report and informant‐report. Each form encompasses nine scales to assess executive functions. The study aimed to examine the unidimensionality of each scale and item‐level agreement between the two forms in people with schizophrenia.

**Methods:**

A total of 197 people with schizophrenia and 194 caregivers were recruited from a single psychiatric center. Among the people with schizophrenia, 44.2% were male, with a mean age of 44.0 years. As for the caregivers who completed the informant‐report form, 27.8% were male, with a mean age of 60.4 years. The unidimensionality of each BRIEF‐A scale was assessed using Rasch analysis. Weighted kappa and percentage of agreement were used to examine the item‐level agreement between the two forms.

**Results:**

The infit and outfit mean squares of all items on each scale of the self‐report form were 0.65–1.48. Except for the Plan/Organize scale, the items of the other eight scales in the informant‐report form met the standards for infit and outfit mean squares (0.69–1.44). Principal component analysis of the standardized residuals of the nine scales were 1.4–1.8 and 1.5–2.1 for the self‐report and informant‐report forms, respectively. Weighted kappa and percentage of agreement between the two forms were 0.02–0.33 and 36.8%–63.4% respectively.

**Conclusion:**

The BRIEF‐A has acceptable unidimensionality for people with schizophrenia. The slight to fair item‐level agreement between the two forms necessitates cautious explanation of results and incorporation of multiple informants' perspectives to ensure a more thorough and accurate assessment of executive functions.

## 1 Introduction

Executive functions are goal‐directed processes involved in guiding, directing, and managing cognitive, emotional, and behavioral functions, such as initiating, planning, self‐control, self‐monitoring, problem solving, and working memory (Kristensen et al. 2024; Tyburski et al. 2021; van Aken et al. 2023). Executive deficits are the primary cognitive impairments in people with schizophrenia (Chiu et al. 2021; Tyburski et al. 2021). Executive deficits affect their ability to maintain independence in daily activities, work, and social participation in the home and community environments (Ruiz‐Castañeda et al. 2020; Tyburski et al. 2021). Therefore, clinicians and researchers must use a measure of executive functions to determine the condition of people with schizophrenia and to design plans for interventions.

The Behavior Rating Inventory of Executive Function‐Adults version (BRIEF‐A) is one of the commonly used measures for assessing executive functions in people with schizophrenia (Haugen et al. 2022; Randers et al. 2020; Raudeberg et al. 2023). It has the following four features: First, it captures the manifestation of executive function difficulties in daily life, thereby providing a practical perspective for clinicians and researchers (Roth et al. 2005). Second, it assesses a wide range of executive functions using nine scales: Inhibit, Shift, Emotional Control, Self‐Monitor, Initiate, Working Memory, Plan/Organize, Task Monitor, and Organization of Materials (Roth et al. 2005). The scales enable a detailed understanding of individuals' strengths and challenges in executive functions. Third, the BRIEF‐A is a questionnaire designed for ease of administration and takes approximately 10–15 min to complete (Raudeberg et al. 2023). Fourth, the questionnaire contains two forms: self‐report and informant‐report. The self‐report form allows individuals to provide their perspectives on their executive function challenges, whereas the informant‐report form gathers observations from informants who are familiar with the individual's daily functioning. The two forms offer clinicians and researchers valuable insights by presenting different perspectives on an individual's executive function performance. If an individual is unable to complete the measure (e.g., language barriers), an assessment from the informant can provide valuable messages (van der Linden et al. 2020). Therefore, the BRIEF‐A is a practical and comprehensive tool for assessing executive functions in people with schizophrenia.

Regarding the psychometric properties of the BRIEF‐A, the construct validity of the informant‐report form has been examined using exploratory factor analysis and confirmatory factor analysis in people with schizophrenia. The results showed that it was a three‐factor model (Power et al. 2012). Each of the nine scales in the BRIEF‐A is intended to assess a specific executive function. However, the unidimensionality of each scale has not been examined in people with schizophrenia. Unidimensionality refers to the concept that a scale assesses a single underlying construct, with all items contributing to the evaluation of that single dimension (Lee et al. 2023). Summing the item scores of each scale is valid if unidimensionality is established. Thus, examining the unidimensionality of each scale in the BRIEF‐A is essential to ensure that clinicians and researchers can apply the sum scores of each scale to represent the specific executive function.

Rasch analysis is a robust method for examining unidimensionality in item‐level analysis (Lin et al. 2025; Wang et al. 2025). Unidimensionality using Rasch analysis of the BRIEF‐A for self‐report and informant‐report forms have not been examined. Moreover, the agreement of responses for each item across the two forms has not been investigated, limiting the explanation and usability of the BRIEF‐A. Therefore, the purposes of this study were to examine the unidimensionality of each scale and item‐level agreement between self‐report and informant‐report forms in people with schizophrenia.

## 2 Methods

### 2.1 Participants

We recruited people with chronic schizophrenia and their caregivers to investigate the unidimensionality of the BRIEF‐A. This study was approved by the research ethics committee of Taipei City Hospital (TCHIRB‐11004008). Written informed consent was obtained from all participants. People with schizophrenia were recruited from a psychiatric center in northern Taiwan between August 2023 and January 2024. The following inclusion criteria were used: (1) diagnosis of schizophrenia according to the Diagnostic and Statistical Manual of Mental Disorders, fifth Edition; (2) ability to read; (3) age > 20 years; and (4) willing to provide informed consent. The exclusion criteria were as follows: (1) history of severe brain injury; (2) diagnosis of substance use disorder or intellectual developmental disorder; and (3) inability to follow the instructions. The inclusion criteria of the caregivers were as follows: (1) main caregiver and living with people with schizophrenia for the past 5 years; (2) age > 20 years; and (3) willing to sign informed consent. Caregivers with mental illness or inability to complete the BRIEF‐A were excluded.

### 2.2 Procedures

Eligible people with schizophrenia were referred by occupational therapists. Two research assistants explained the study to patients with schizophrenia and their caregivers individually. After obtaining written informed consent, the participants with schizophrenia and their caregivers completed the BRIEF‐A self‐report and informant‐report, respectively. The demographic data of participants with schizophrenia were collected from medical records.

### 2.3 Measures

The BRIEF‐A assesses executive functions using nine scales with 70 items. The Inhibit scale (eight items) assesses an individual's ability of inhibitory control (i.e., resist, suppress, or refrain from acting on an impulse) and the ability to halt one's behavior at the appropriate moment. The Shift scale (six items) assesses an individual's ability to smoothly transition between different situations, activities, or aspects of a problem, as per the situation. The Emotional Control scale (10 items) assesses an individual's ability to regulate emotional responses. The Self‐Monitor scale (six items) assesses an individual's ability to monitor one's behavior and the impact of that behavior on others. The Initiate scale (eight items) includes items that focus on starting a task and generating ideas, responses, or solutions to problems independently. The Working Memory scale (eight items) assesses an individual's ability to actively retain information to complete a task or generate a response. The Plan/Organize scale (10 items) assesses an individual's ability to manage current and future task demands in a given situational context. The Task Monitor scale (six items) assesses an individual's ability to monitor task performance, specifically, tracking one's success or failure in solving problems. The Organization of Materials scale (eight items) assesses an individual's ability to maintain order and organization in one's daily environment, including work, living, and storage spaces. Each item of the BRIEF‐A is rated on three‐points: 1 = never, 2 = sometimes, and 3 = often. The sum scores of each scale represents the specific executive function, with higher scores demonstrating worse scale‐specific function and vice versa (Roth et al. 2005).

### 2.4 Data Analysis

Rasch analysis was performed to investigate the unidimensionality of each scale for both forms of the BRIEF‐A. The Rasch rating scale model was developed using WINSTEPS. Infit and outfit mean squares (MnSq) were applied to confirm whether the item responses met the expectations of the unidimensional Rasch model for each scale. An item was considered a misfit if its infit or outfit MnSq value was < 0.5 or > 1.5 (Arisanti et al. 2020; Lee et al. 2023). Moreover, unidimensionality was verified using principal component analysis (PCA) of standardized residuals. We computed the eigenvalue of the residual variance for the first contrast. Unidimensionality was met if eigenvalue was < 3.0 (Chiu et al. 2019).

Weighted kappa and percentage of agreement were applied to investigate item‐level agreement between the two forms of the BRIEF‐A. The criterion of weighted kappa was as follows: ≤ 0.20, slight agreement; 0.21–0.40, fair agreement; 0.41–0.60, moderate agreement; 0.61–0.80, good agreement; and 0.81–1.00, excellent agreement (Sim and Wright 2005). A higher percentage of agreement reflected better item‐level agreement between the two forms.

## 3 Results

A total of 197 people with chronic schizophrenia completed the BRIEF‐A self‐report form. Approximately 44.2% of the participants with schizophrenia were male and the mean age was 44.0 years. The mean duration since onset of schizophrenia was 21.9 years. The demographics of the participants with schizophrenia are presented in Table 1. The informant‐report form was completed by 194 caregivers. Among them, 54 were male (27.8%), and the mean age (standard deviation) was 60.4 years (15.2).

**TABLE 1:** Demographics of participants with schizophrenia (*n* = 197).

Characteristic	
Gender (%)	
Male	87 (44.2)
Female	110 (55.8)
Age (mean year [SD])	44.0 (11.7)
Onset age (mean year [SD])	21.9 (8.1)
Education (%)	
Elementary school	6 (3.0)
Junior high school	17 (8.6)
Senior high school	83 (42.1)
College and above	91 (46.2)
Type of antipsychotics (%)	
First generation	10 (5.1)
Second generation	102 (51.8)
Third generation	18 (9.1)
Taking two types	41 (20.8)

Abbreviation: SD = standard deviation.

The infit and outfit statistics of the nine scales in the self‐report form met the expectations of the unidimensional Rasch model (0.65–1.48) (Table 2). The residuals of PCA on the Inhibit, Shift, Emotional Control, Self‐Monitor, Initiate, Working Memory, Plan/Organize, Task Monitor, and Organization of Materials scales were 1.6, 1.4, 1.8, 1.8, 1.5, 1.6, 1.8, 1.7, and 1.7, respectively. For the informant‐report form, the outfit MnSq of Item 21 in the Plan/Organize scale was higher than 1.5 (Table 2). The items of the other eight scales met the standards for infit and outfit MnSqs (0.69–1.44). The residuals of PCA were 1.8, 1.8, 1.6, 1.5, 1.7, 1.5, 1.7, 1.7, and 2.1 in the Inhibit, Shift, Emotional Control, Self‐Monitor, Initiate, Working Memory, Plan/Organize, Task Monitor, and Organization of Materials scales, respectively.

**TABLE 2:** Results of unidimensionality using Rasch analysis and item‐level agreement.

Scale	Item number	Self‐report form			Informant report form			Weighted kappa	Percentage of agreement
		Item difficulty	Infit MnSq	Outfit MnSq	Item difficulty	Infit MnSq	Outfit MnSq		
Inhibit	5	0.18	1.36	1.48	0.46	1.40	1.42	0.25	53.6
	16	0.13	0.94	0.92	0.03	1.17	1.18	0.25	48.7
	29	−0.09	1.02	1.07	0.19	0.87	0.83	0.20	47.7
	36	0.72	0.85	0.94	0.66	1.06	0.98	0.15	57.6
	43	−0.09	0.91	0.85	0.10	0.98	1.02	0.21	52.1
	55	−0.31	0.92	0.97	−0.71	0.75	0.84	0.25	49.0
	58	−0.15	1.09	1.16	−0.66	0.85	0.85	0.19	45.9
	73	−0.38	0.89	0.83	−0.06	0.95	0.94	0.17	45.4
Shift	8	0.04	1.27	1.34	0.42	0.94	0.91	0.14	44.3
	22	0.49	0.84	0.81	0.04	1.02	0.99	0.12	44.0
	32	−0.25	1.03	1.03	−0.71	1.02	1.05	0.02	37.8
	44	−0.06	0.94	0.90	−0.06	0.89	0.87	0.16	44.8
	61	−0.03	1.04	1.05	0.71	1.08	1.03	0.27	53.1
	67	−0.18	0.86	0.84	−0.40	1.00	0.92	0.23	49.0
Emotional control	1	0.05	1.28	1.41	1.59	0.82	0.73	0.27	60.1
	12	−0.37	0.93	0.90	−0.02	0.91	0.87	0.29	51.3
	19	1.43	1.15	0.90	1.67	0.97	0.83	0.28	63.4
	28	0.79	0.89	0.88	0.17	0.92	0.88	0.29	56.0
	33	−0.01	1.02	0.95	−1.05	1.23	1.23	0.19	42.3
	42	−0.97	0.71	0.74	−1.23	0.90	0.86	0.33	53.1
	51	−0.37	1.31	1.43	0.08	1.43	1.38	0.15	47.4
	57	0.15	0.94	0.86	0.20	0.84	0.75	0.27	52.8
	69	−0.31	0.86	0.80	−0.11	0.85	0.81	0.30	51.5
	72	−0.40	0.92	0.90	−1.31	1.03	1.01	0.16	44.6
Self‐monitor	13	−0.10	1.07	1.08	0.18	1.21	1.21	0.15	42.5
	23	0.49	0.91	0.89	0.44	1.36	1.40	0.15	49.0
	37	−0.27	0.88	0.88	−0.41	0.86	0.86	0.19	48.0
	50	−0.22	1.14	1.10	−0.03	0.87	0.85	0.21	41.5
	64	0.08	1.02	1.02	−0.03	0.90	0.88	0.28	48.7
	70	0.02	0.94	0.94	−0.16	0.78	0.74	0.18	48.0
Initiate	6	−0.32	1.05	1.10	−0.12	0.89	0.92	0.14	44.8
	14	0.55	0.69	0.65	1.02	1.08	1.07	0.18	50.0
	20	0.18	1.22	1.27	−0.62	1.17	1.22	0.09	39.3
	25	−0.05	1.04	0.99	−0.76	0.68	0.64	0.16	39.2
	45	−0.05	1.13	1.17	0.08	1.21	1.21	0.12	41.5
	49	−0.03	0.84	0.81	0.35	0.77	0.76	0.27	49.5
	53	0.04	1.03	0.99	0.57	1.19	1.19	0.28	49.0
	62	−0.32	0.99	1.00	−0.52	0.98	0.96	0.18	39.2
Working memory	4	0.20	1.11	1.07	−0.54	0.95	0.92	0.09	42.0
	11	0.29	0.99	1.06	0.30	0.84	0.80	0.12	48.5
	17	0.48	0.88	0.90	1.39	1.25	1.16	0.30	59.8
	26	0.48	0.92	0.97	0.35	1.13	1.06	0.17	46.4
	35	−0.48	1.01	0.99	−0.52	0.85	0.90	0.26	46.1
	46	−0.04	0.70	0.71	−0.35	0.89	0.87	0.10	46.4
	56	−0.02	1.06	1.06	1.21	1.14	1.26	0.15	46.9
	68	−0.90	1.29	1.29	−1.83	0.98	0.93	0.28	44.8
Plan/Organize	9	−0.82	1.25	1.45	−0.95	1.04	1.28	0.07	36.8
	15	0.08	0.75	0.76	0.24	0.77	0.78	0.11	39.7
	21	0.52	1.14	1.13	1.07	1.37	1.53	0.21	52.6
	34	−0.19	1.23	1.19	−0.65	0.90	0.85	0.22	40.4
	39	0.14	1.27	1.38	0.32	1.04	1.11	0.20	46.6
	47	−0.11	1.04	1.02	0.01	1.41	1.39	0.11	42.5
	54	0.31	0.70	0.71	0.84	0.99	1.05	0.16	53.6
	63	−0.17	0.97	0.97	−0.59	0.71	0.77	0.30	48.5
	66	0.14	0.82	0.81	−0.18	0.91	0.86	0.22	43.8
	71	0.10	0.79	0.75	−0.11	0.78	0.77	0.17	44.3
Task monitor	2	−0.87	1.21	1.25	−0.32	0.82	0.83	0.21	53.1
	18	0.49	1.09	1.04	−0.47	0.96	0.95	0.24	47.2
	24	−0.01	0.81	0.79	−0.97	1.03	1.05	0.19	46.4
	41	−0.43	1.03	1.05	0.00	1.02	1.05	0.16	41.8
	52	0.62	0.91	0.85	0.90	1.14	1.22	0.19	44.3
	75	0.19	0.93	0.86	0.86	0.99	0.91	0.15	47.4
Organization of materials	3	−0.30	1.00	1.01	−0.83	0.98	1.04	0.17	40.9
	7	−0.84	1.25	1.26	−0.85	1.00	0.95	0.23	49.2
	30	0.41	0.96	1.09	−0.71	1.00	0.98	0.16	42.8
	31	0.10	1.08	1.12	0.63	1.32	1.44	0.27	52.1
	40	0.66	0.87	0.89	1.28	1.25	1.11	0.24	57.7
	60	−0.34	0.92	0.94	0.05	0.82	0.78	0.22	52.1
	65	−0.06	0.79	0.83	0.42	0.98	0.88	0.22	52.6
	74	0.36	1.07	1.02	0.01	0.70	0.69	0.15	46.9

Abbreviation: MnSq = mean square.

Weighted kappa of the 70 items between two forms ranged from 0.02 to 0.33 (Table 2). The percentages of agreement for the 70 items were 36.8%–63.4%.

## 4 Discussion

This is the first study to examine the unidimensionality of self‐report and informant‐report forms and item‐level agreement between two forms of the BRIEF‐A for people with schizophrenia. According to the results of the Rasch analysis and residuals in the PCA, the unidimensionality of the scales in the two forms was supported, except for the Plan/Organize scale of the informant‐report form. This study showed slight to fair item‐level agreement between the two forms. The findings of this study are essential for clinicians and researchers to effectively explain and utilize the two forms in people with schizophrenia.

Rasch analysis showed that all nine scales of the self‐report form and eight out of the nine scales of the informant‐report form demonstrated satisfactory unidimensionality in people with schizophrenia. That is, the items within each scale can be summed to represent scale‐specific executive function. These findings are partially consistent with a previous study using confirmatory factor analysis in healthy adults, which demonstrated unidimensionality of all nine scales in both the self‐report and informant‐report forms (Albuquerque et al. 2023). For the informant‐report form, only one item (Item 21) of the Plan/Organize scale showed poor‐fitting in outfit MnSq (> 1.5), which demonstrates that responses from the informants deviated from the expected pattern, such as random guessing or inconsistent answering (Chiu et al. 2015). The poor‐fit of Item 21 (“starts tasks [such as cooking, projects] without the right materials”) may be because informants may not have sufficient opportunities to observe people with schizophrenia engaging in tasks like cooking or projects, leading to less accurate or less informed responses. Item 21 reflects the ability to plan to attain goals and implement the plans effectively, which are critical components of executive functions (Chiu et al. 2021). The residuals of the PCA on the Plan/Organize scale (1.7) met the criteria (< 3.0). Thus, Item 21 of the informant‐report form was retained. Future research could involve professionals (e.g., occupational therapists) who provide support in activities of daily living and work training. Their responses to the informant‐report form could further validate unidimensionality.

According to the results of weighted kappa (≥ 0.21) and percentage of agreement (> 50%), 17 items of 7 scales showed fair agreement between two forms in the BRIEF‐A, including 2, 1, 7, 1, 1, 1, and 4 items in the Inhibit, Shift, Emotional Control, Working Memory, Plan/Organize, Task Monitor, and Organization of Materials scales, respectively. Compared to other scales, the Emotional Control scale demonstrated relatively higher agreement, with seven out of 10 items showing fair consistency between the self‐report and informant‐report forms. This may be because emotional control involves observable behaviors, such as angry outbursts (Item 1) and getting emotionally upset easily (Item 42).

Items 9 (get overwhelmed by large tasks) and 32 (have trouble thinking of a different way to solve a problem when stuck) had relatively lower weighted kappa and percentage of agreement among the 70 items, likely due to the subjective and context‐dependent nature of these behaviors. Informants (caregivers in this study) may lack sufficient opportunities to observe responses to task complexity and cognitive flexibility in real‐time among people with schizophrenia. A previous study stated that self‐reports of everyday functioning by people with schizophrenia showed minimal correlation with informants' reports (Knížková et al. 2023). Although the item‐level agreement was limited, caregivers' perspectives offered critical awareness of behaviors and daily functioning related to executive functions in people with schizophrenia. To improve item‐level agreement between people with schizophrenia and their caregivers, future research could utilize structured observational methods (e.g., specific task scenarios) that allow caregivers to witness and assess these behaviors in controlled environments.

Four limitations were noted in this study. First, we recruited people with schizophrenia from only one psychiatric center, restricting the generalizability of the results. Further studies with samples of schizophrenia from other facilities are required to cross‐examine the findings. Second, limited demographic information (i.e., gender and age) was collected from caregivers, which may restrict the interpretation of findings related to informant‐reported responses. Third, caregivers of people with schizophrenia were invited to fill the informant‐report form, which may not fully capture the functional abilities of people with schizophrenia in contexts outside the caregiver's observation, such as in rehabilitation settings. Further studies on the perspective of other informants (e.g., occupational therapists) are warranted. Fourth, the test‐retest reliability of the BRIEF‐A has not been investigated in people with schizophrenia, which may restrict the interpretation of the test results. Further studies are recommended to investigate the test‐retest reliability of the BRIEF‐A.

## 5 Conclusion

Our findings provide empirical evidence to support the unidimensionality of the nine scales in the self‐report form and eight scales in the informant‐report form of the BRIEF‐A for people with schizophrenia. The finite item‐level agreement between the two forms highlights the need for careful explanation of the results and suggests integrating multiple informants' perspectives to achieve a broader and more accurate assessment of executive functions.

## Author Contributions

**En‐Chi Chiu:** conceptualization, formal analysis, writing – original draft preparation. **Ya‐Chen Lee:** formal analysis, writing – review and editing. **Yi‐Ching Wu:** investigation. **Shu‐Chun Lee:** conceptualization, data curation, funding acquisition, writing – review and editing.

## Conflicts of Interest

The authors declare no conflicts of interest.

## Data Availability

The data that support the findings of this study are available from the corresponding author upon reasonable request.
